# Parental education, cognition and functional connectivity of the salience network

**DOI:** 10.1038/s41598-023-29508-w

**Published:** 2023-02-16

**Authors:** Pavla Cermakova, Adam Chlapečka, Zsófia Csajbók, Lenka Andrýsková, Milan Brázdil, Klára Marečková

**Affiliations:** 1 Second Faculty of Medicine, Charles University Prague, 150 06 Prague 5, Czech Republic; 2 National Institute of Mental Health, 250 67 Klecany, Czech Republic; 3 Third Faculty of Medicine, Charles University Prague, 100 00 Prague 10, Czech Republic; 4 Centre of Clinical Neuroscience, Department of Neurology, First Faculty of Medicine, General University Hospital, Charles University in Prague, 128 21 Prague 2, Czech Republic; 5 Faculty of Humanities, Charles University Prague, 182 00 Prague 8, Czech Republic; 6 RECETOX, Faculty of Science, Masaryk University, 611 37 Brno, Czech Republic; 7 Brain and Mind Research, Central European Institute of Technology, Masaryk University, 625 00 Brno, Czech Republic

**Keywords:** Risk factors, Neuroscience, Development of the nervous system, Neural circuits, Biomarkers, Epidemiology

## Abstract

The aim was to investigate the association of parental education at birth with cognitive ability in childhood and young adulthood and determine, whether functional connectivity of the salience network underlies this association. We studied participants of the Czech arm of the European Longitudinal Study of Pregnancy and Childhood who underwent assessment of their cognitive ability at age 8 (Wechsler Intelligence Scale for Children) and 28/29 years (Wechsler Adult Intelligence Scale) and measurement with resting state functional MRI at age 23/24. We estimated the associations of parental education with cognitive ability and functional connectivity between the seeds in the salience network and other voxels in the brain. We found that lower education of both mothers and fathers was associated with lower verbal IQ, performance IQ and full-scale IQ of the offspring at age 8. Only mother´s education was associated with performance IQ at age 28/29. Lower mother´s education correlated with greater functional connectivity between the right rostral prefrontal cortex and a cluster of voxels in the occipital cortex, which, in turn, was associated with lower performance IQ at age 28/29. We conclude that the impact of parental education, particularly father´s, on offspring´s cognitive ability weakens during the lifecourse. Functional connectivity between the right rostral prefrontal cortex and occipital cortex may be a biomarker underlying the transmission of mother´s education on performance IQ of their offspring.

## Introduction

High cognitive ability is one of the strongest predictors of healthy and successful lives^1,2^. Besides a strong genetic component, cognitive ability can be to a great extent enhanced by stimulating early-life environment^3^. Educational attainment of parents is a commonly used proxy for the quality of early-life socioeconomic and cognitively stimulating environment^4^ and is strongly tied to the child´s later cognition^5^. Both father´s as well as mother´s education has been shown to be associated with cognitive ability of their offspring^6^, but the evidence varies as to whether the education of the mother or the father has greater impact on cognition of the children, depending on the country, time as well as type of cognitive ability studied^7–9^. For example, in a study of intelligence quotient (IQ) across generations using a British cohort, fathers' education better predicted children's IQ differences in the 1946 cohort, but in the latest cohort from 2000 to 2002, mothers' education was more strongly associated with children's IQ^10^.

The mechanisms underlying the association of parental education with offspring´s cognitive ability are still unclear. Cognitive ability has been proposed to be the result of large-scale brain networks^11^ and an increasing body of evidence points towards alterations in their resting state functional connectivity as the potential neural underpinning of interindividual differences in cognitive ability^12^. Parallel to this, there have been efforts to describe the impact of early-life socioeconomic exposures on alterations of the brain networks. For example, Gellci et al. suggested that childhood socioeconomic disadvantage impacts the development of the salience network^13^. Specifically, they found that childhood socioeconomic disadvantage was associated with less efficient global information transfer and fewer connections of the anterior cingulate cortex and the left supramarginal gyrus^13^. Moreover, greater childhood socioeconomic disadvantage was associated with lower IQ, which, in turn, correlated with global efficiency of the left supramarginal gyrus, where global efficiency measured the extent of information transmission of the left supramarginal gyrus with all other regions of interest within the salience and emotion network^13^. Alterations in the functional connectivity of the salience network were also found correlated with the quality of maternal behavior^14^, parent-childhood communication^15^ and childhood abuse and neglect^16^, which are all factors strongly associated with early-life socioeconomic environment, including parental education^17^.

The salience network has been previously found associated with several measures of cognitive ability^18,19^ and is thought to mediate information flow between cognitive and emotional systems, select stimuli that are behaviorally relevant and coordinate neural reponse towards them. It is, therefore, plausible to hypothesize that the effects of parental education may be mirrored in the alterations in the functional connectivity of the salience network of their offspring as well as in their cognitive ability. Capitalizing on participants from a birth cohort that have been followed-up for almost 30 years, we aimed to determine the association of parental education with cognitive ability in childhood and young adulthood and investigate, whether functional connectivity of the salience network underlies this association.

## Results

### Parental education and cognitive ability

Lower mother´s education was associated with lower cognitive ability at age 8 measured by verbal IQ (Spearman´s rho − 0.322; *p* < 0.001), performance IQ (Spearman´s rho − 0.245; *p* < 0.001) and full-scale IQ (Spearman´s rho − 0.322; *p* < 0.001). The association persisted when adjusted for sex, father´s education, father´s occupation and household socioeconomic situation (verbal IQ: B = − 1.797; 95% confidence interval [CI] − 2.620 to − 0.973; performance IQ: B = − 1.088; 95% CI − 1.970 to − 0.205; full scale IQ: B = − 1.603; 95% CI − 2.429 to − 0.776; Table 1). Similarly, lower father´s education was associated with lower cognitive ability measured by verbal IQ (Spearman´s rho − 0.344; *p* < 0.001), performance IQ (− 0.271; *p* < 0.001) and full-scale IQ (Sperman´s rho − 0.356; *p* < 0.001). The association persisted when adjusted for sex, mother´s education, father´s occupation and household socioeconomic situation (verbal IQ: B = − 1.347; 95% CI − 2.312 to − 0.381; performance IQ: B = − 1.405; 95% CI − 2.440 to − 0.370; full scale IQ: B = − 1.434; 95% CI − 2.401 to − 0.466). In the fully adjusted model, mother´s education had a slightly stronger association with verbal IQ (mother´s education: β = − 0.207; father´s education: β = − 0.178), but a slightly weaker association with performance IQ (mother´s education: β = − 0.121; father´s education: β = − 0.179).

**Table 1:** Associations of parental education with cognitive ability at age 8.

	B (95% CI)	Β	*p* value
Performance IQ			
Mother´s education	− **1.088 (**− **1.970; -0.205)**	− **0.121**	**0.016**
Father´s education	− **1.405 (**− **2.440; -0.370)**	− **0.179**	**0.008**
Sex (males)	− 0.761 (− 3.513; 1.992)	− 0.022	0.588
Father´s occupation	0.154 (− 0.668; 0.977)	0.023	0.713
Household socioeconomic circumstances	− 0.138 (− 2.777; 2.501)	− 0.004	0.918
Verbal IQ			
Mother´s education	− **1.797 (**− **2.620; **− **0.973)**	− **0.207**	**< 0.001**
Father´s education	− **1.347 (**− **2.312; **− **0.381)**	− **0.178**	**0.006**
Sex (males)	0.046 (− 0.522; 2.614)	0.001	0.972
Father´s occupation	− 0.018 (− 0.785; 0.750)	− 0.003	0.964
Household socioeconomic circumstances	− 1.200 (− 3.662; 1.261)	− 0.039	0.339
Full scale IQ			
Mother´s education	− **1.603 (**− **2.429; **− **0.776)**	− **0.185**	**< 0.001**
Father´s education	− **1.434 (**− **2.401; **− **0.466)**	− **0.190**	**0.004**
Sex (males)	− 0.442 (− 3.019; 2.134)	− 0.013	0.736
Father´s occupation	− 0.036 (− 0.804; 0.733)	− 0.006	0.928
Household socioeconomic circumstances	− 1.213 (− 3.688; 1.263)	− 0.040	0.336

IQ = intelligence quotient; B = unstandardized coefficient from linear regression; β = standardized coefficient from linear regression; CI = confidence interval. All variables are included in the models.

Significant values are in [bold].

When assessing cognitive ability at age 28/29, lower mother´s education was associated with lower performance IQ (Spearman´s rho − 0.251; *p* = 0.021) and full-scale IQ (Spearman´s rho − 0.224; *p* = 0.041), but not with verbal IQ (Spearman´s rho − 0.050; *p* = 0.652). The association to performance IQ persisted when adjusted for sex, father´s education, father´s occupation and household socioeconomic situation (B = − 2.276; 95% CI − 4.506 to − 0.046), but the association to full-scale IQ lost statistical significance (Table 2). Father´s education was not associated with any measure of cognitive ability at age 28/29. We did not detect any effect modification by sex in any model (*p* from likelihood ratio test > 0.05 in all models).

**Table 2:** Associations of parental education with cognitive ability at age 28/29.

	B (95% CI)	Β	*p* value
Performance IQ			
Mother´s education	**− 2.276 (**− **4.506; **− **0.046)**	− **0.238**	**0.046**
Father´s education	− 0.879 (**− **3.846; 2.089)	− 0.102	0.557
Sex (males)	3.132 (**− **5.048; 11.313)	0.085	0.448
Father´s occupation	0.324 (− 2.149; 2.798)	0.046	0.795
Household socioeconomic circumstances	0.773 (− 6.245; 7.790)	0.025	0.827
Full scale IQ			
Mother´s education	− 1.542 (− 3.288; 0.204)	− 0.207	0.083
Father´s education	− 1.245 (− 3.569; 1.079)	− 0.185	0.289
Sex (males)	− 1.548 (− 7.954; 4.858)	− 0.054	0.632
Father´s occupation	0.423 (− 1.514; 2.360)	0.077	0.665
Household socioeconomic circumstances	− 0.347 (− 5.843; 5.148)	− 0.015	0.900

IQ = intelligence quotient; B = unstandardized coefficient from linear regression; β = standardized coefficient from linear regression; CI = confidence interval. All variables are included in the models.

Significant values are in [bold].

### Functional connectivity of the salience network

Reflecting the results, we focused on the investigation of the functional connectivity underlying the association of mother´s education with performance IQ at age 28/29. Mother´s education was associated with functional connectivity between 4 nodes of the salience network (right anterior insula, left rostral prefrontal cortex (PFC), right rostral PFC and left supramarginal gyrus) and in total 7 different clusters (see Table 3). Specifically, lower mother´s education correlated with greater connectivity between the right anterior insula and cluster 1 (right intracalcarine cortex and lingual gyrus, false discovery rate (FDR) corrected *p* = 0.025); between the left rostral PFC and cluster 2 (left parts of the lateral occipital cortex, FDR-*p* = 0.021) and between the right rostral PFC and cluster 3 (several regions in the occipital cortex, FDR-*p* < 0.001). In addition, lower mother´s education was associated with greater connectivity between the left supramarginal gyrus and four different clusters (clusters 4–7): Cluster 4 included regions in the temporal lobe and cerebellum (FDR-*p* = 0.001); cluster 5 parts of the right division of the lateral occipital cortex (FDR-*p* = 0.007); cluster 6 parts of the left division of the lateral occipital cortex (FDR-*p* = 0.025); and cluster 7 parts of the lateral occipital cortex and precuneus cortex (FDR-*p* = 0.025).

**Table 3:** Clusters of voxels functionally connected with nodes of salience network, correlating with mother´s education.

Cluster	MNI (x, y, z)	Size	*p*-FWE	*p*-FDR	Regions
Seed: Right anterior insula					
1	+ 24; − 68; − 04	188	0.032	0.025	Intracalcarine cortex right; lingual gyrus right
Seed: Left rostral prefrontal cortex					
2	− 48; − 82; + 22	245	0.011	0.021	Lateral occipital cotex, superior division left; lateral occipital cortex, inferior division left
Seed: Right rostral prefrontal cortex					
3	+ 14; -88; − 06	744	< 0.001	< 0.001	Occipital pole right; lingual gyrus right; lingual gyrus left; intracalcarine cortex right; intracalcarine cortex left; occipital pole left; occipital fusiform gyrus right; cuneal cortex right; supracalcarine cortex right
Seed: Left supramarginal gyrus					
4	− 50; − 54; − 16	433	< 0.001	0.001	Inferior temporal gyrus left; inferior temporal gyrus, temporooccipital part left; temporal occipital fusiform cortex left; hippocampus left; cerebelum 6 left; cerebelum crus1 left; cerebelum 8 left; cerebelum 7b left; middle temporal gyrus, temporooccipital part left
5	+ 50; − 78; + 22	276	0.006	0.007	Lateral occipital cortex, superior division right; lateral occipital cortex, inferior division right
6	− 46; − 80; + 24	184	0.041	0.025	Lateral occipital cortex, superior division left; lateral occipital cortex, inferior division left
7	+ 10; − 72; + 60	177	0.047	0.025	Lateral occipital cortex, superior division right; precuneous cortex

FWE: family-wise error; FDR: false discovery rate; MNI: Montreal Neurological Institute.

**Figure 1:**
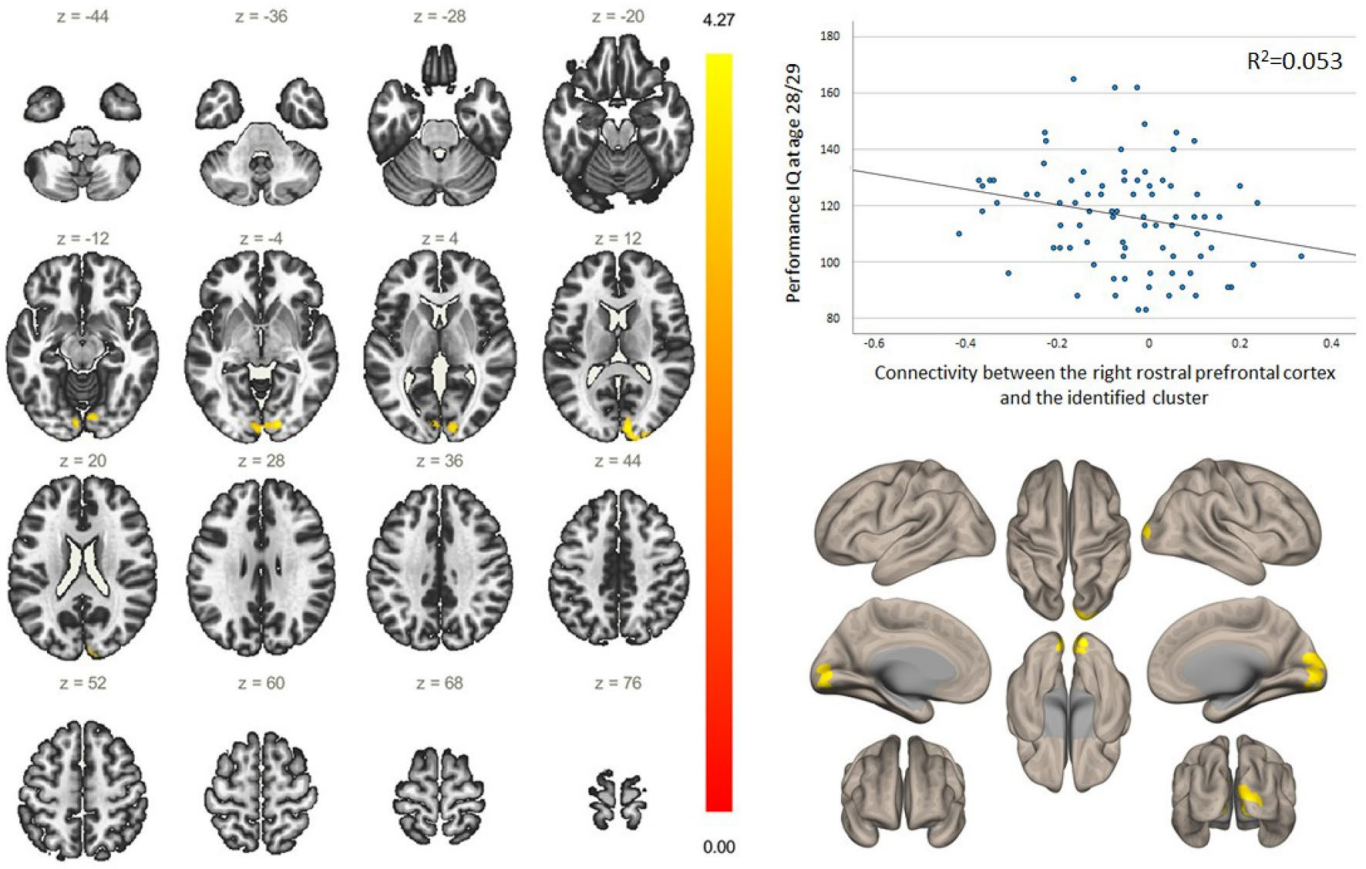
Functional connectivity of the right rostral prefrontal cortex and its relation to cognitive ability. Figures on the left and bottom right demonstrate in yellow a cluster of voxels (MNI + 14; − 88; − 06) correlating with the right rostral prefrontal cortex. The cluster includes right and left occipital pole, right and left lingual gyrus, right and left intracalcarine cortex, right occipital fusiform gyrus, right cuneal cortex and right supracalcarine cortex.

Greater performance IQ correlated with lower connectivity between three nodes and in total three clusters when uncorrected *p* value was used, however, only one association survived the Benjamini–Hochberg correction that adjusted the p value due to the testing of the 7 clusters (Spearman´s rho − 0.267; FDR-*p* = 0.049). Specifically, it was the association with the connectivity between the right rostral PFC and cluster 3 that included the right and left occipital pole, right and left lingual gyrus, right and left intracalcarine cortex, right occipital fusiform gyrus, right cuneal cortex and right supracalcarine cortex (Fig. 1).

In secondary analysis, we additionally explored whether the connectivity between the right rostral PFC and the aforementioned cluster 3 is associated with other measures of cognitive ability at age 28/29 as well as age 8. We found that greater full-scale IQ at age 28/29 was associated with lower connectivity between the right rostral PFC and this cluster (sex-adjusted, B = − 0.003; 95% CI − 0.005, − 0.0003; uncorrected *p* value = 0.029), but was not related to verbal IQ at this age (sex-adjusted, B = − 0.001; 95% CI − 0.004, 0.001; uncorrected *p* value = 0.183). Interestingly, at age 8, greater cognitive ability irrespective of measurement was associated with lower connectivity between the right rostral PFC and this cluster: B = − 0.004; 95% CI − 0.007, − 0.001, *p* = 0.013 for verbal IQ; B = − 0.003; 95% CI − 0.006, − 0.0001, *p* = 0.046 for performance IQ; B = − 0.004; 95% CI − 0.006, − 0.001, *p* = 0.008 for full scale IQ (associations are sex-adjusted and p values uncorrected).

## Discussion

In the current study, capitalizing on data from participants of a prenatal birth cohort that have been followed for almost 3 decades, we found that education of both mothers and fathers predicted cognitive ability (performance IQ, verbal IQ, and full-scale IQ) in offspring aged 8 years. However, in offspring aged 28/29 years, father´s education was not associated with any domain of cognitive ability, and mother´s education only with performance IQ. Greater education of mothers as well as greater performance IQ of the offspring at age 28/29 were associated with lower functional connectivity between the right rostral PFC, a node in the salience network, and a cluster of voxels situated mainly in the occipital cortex.

A robust body in literature indicates the transmission of human capital from parents to children. For example, a positive relationship between mother´s education and academic outcome of their children has been established^20^. Studies found a connection between increasing educational level of young, low-educated mothers with higher cognitive scores of their children^21,22^. Cognitive ability has been shown to be promoted by parental education throughout the whole child development, starting from supporting language skills in early childhood^23^, increasing educational achievement throughout elementary, middle and high school^20,24^ up to university education and occupational success in the productive age^25^. Several mechanisms have been found to explain the relationship between parental education and cognitive ability of their offsprings. For example, highly educated parents use more complex language at home^9,26^, develop more frequently various cognitively stimulating parenting practices, such as teaching their children numbers and letters^27^, or possess higher numbers of learning materials in their homes^28^. These findings can be further supported by rodent models, which reveal that rats housed in enriched environment demonstrate better cognitive, motor, visuospatial and temporal functions compared to standard-housed rats^29^. Furthermore, better compensation of neurodeficits induced by brain lesions and resistance to neurodegeneration was shown in rodents housed in complex environment^30^.

Some research indicates that mother’s education may play a noticeably stronger role in cognitive development of the offspring compared to father’s education^7,28^. A study by Neiss and Rove^31^, which estimated genetic and environmental influences on verbal intelligence, found a stronger mother–child correlation, relative to father-child correlation, in biological families. A commonly suggested mediator in the association of childhood socioeconomic position and cognitive ability of the offspring is the quality and complexity of maternal speech^9,26^. On the other hand, a twin study, which focused on intergenerational schooling, did not show significant positive associations between mother´s education and children’s attainment after controlling for fathers’ education^8^.

Our study complements previous findings that revealed that the impact of both genetic and family environment tends to decline over the life course^32^. We have shown that the impact of mother´s and father´s education is similar on cognitive ability in childhood, but the effects of parental education may decline when one grows up. Specifically, we found that father´s education may even lose its impact on cognitive ability in young adulthood. On the contrary, the effects of mother´s education persist until the third decade, but not in all cognitive domains, and are weaker than in childhood. As the study participants are members of a Czech birth cohort and were born at the beginning of the 1990´s, when the traditional setup of childcare was a long maternity leave lasting 3 years, we argue that our findings may be explained by the more intensive contact that the participants had with their mothers during their development, which possibly left a stronger imprint on their cognitive ability.

While the notion that several markers of socioeconomic status predict general cognitive ability is well established^33,34^, fewer studies have examined the biomarkers of this association. Potential biomarkers could be differences in cortical thickness and cortical volume^35^, whole-brain connectivity patterns^36^ or volume of temporo-parietal white matter clusters^37^. Our study provides novel insights into functional biomakers, specifically suggesting that functional connectivity between the right rostral PFC, a node in the salience network, and a cluster of voxels situated in the occipital cortex may explain how maternal education impacts performance IQ of the adult offspring. Rostral PFC is a structure involved in working memory, episodic memory, multiple-task coordination^38^ and decision-making, prior to the decision being available to consciousness^39^. This area has a particularly high number of dendritic spines per cell in humans^40^, suggesting that high computational power of rostral PFC could be responsible for the integration and coordination of inputs that are particularly developed in human brains^41^. Patients with rostral PFC lesions may have a generally preserved IQ and memory, however, they are often impaired in multitasking, and in situations with multiple possible scenarions, where the most advantageous outcome is not immediately evident^40^.

A previous magnetic resonance imaging (MRI) study showed a rapid increase in the cortical thickness of the rostral PFC in a group of children with high IQ, as compared to average IQ, which peaks at age 13 and wanes in late adolescence^40^. Previous research has also suggested that rostral PFC is connected primarily to other higher-order association cortex areas particularly in other prefrontal regions, while having few connections to primary somatic or motor regions. A study on Macaque monkeys suggested that the lateral stream of fibers, which link rostral PFC to the superior temporal regions, may be responsible for the integration of the auditory and multisensory stimulation, thus permitting control over the most integrated aspects of cognitive processing^42^.

Our finding concerning the connectivity between rostral PFC and areas in the occipital cortex is surprising, as occipital cortex is a well-recognised visual area^43^. As all the structures involved in the identified cluster play a crucial role in visual data processing, we argue that similarly to the pathways between rostral PFC and superior temporal regions involved in the integration of auditory information, connections between rostral PFC and the cluster could be pivotal in the visual processing. Consequently, as discussed before, high computation power of rostral PFC could be used for the integration of the multisensory information as a key aspect of the cognitive processing. The occipital cortex is a composite integrative system, with a ventral stream, which is believed to mainly subserve discrimination of visual shapes and objects, and a dorsal stream that processes spatial information, motion and visually-guided grasping and reaching^44^. Cognitive functions are undoubtedly affected by several aspects of visual processing, including visual working memory and executive attention. In favour of this argument, it is well-documented that patients with Huntington disease, an inherited neurodegenerative disorder, even in the early stages of the disease, present with specific impairment in visual cognition and cognitive performance, which are associated with both structural and functional deficits in occipital visual cortex^45,46^.

Our study indicates that lower functional connectivity between rostral PFC and the aforementioned cluster is reflected in higher cognitive ability. This may seem counterintuitive first when one expects greater connectivity to be linked to better performance, but several works have already shown that “greater is not always better”. In fact, smaller positive or negative changes in functional connectivity may result in optimisation for more efficient network updates, reducing processing demands and energy consumption in order to improve the behavioural performance^47,48^. These results are in line with a well-evidenced process of functional segregation of the networks, which is crucial in a neurodevelopmental period of adolesence and young adulthood^49–51^. Previous research demonstrates that lower functional connectivity between mostly primary unimodal areas and attentional networks is associated with better cognitive flexibility in puberty^52^. On the contrary, this pattern was inverse between attention and task control networks, where stronger connectivity was associated with better cognitive results. Our findings support the evidence that segregation of primary unimodal areas (visual cortex) and higher-order attentional systems (salience network) is a key part of cognitive development. Another explanation of our results could be the process of network maturation, since one of the important features of this process is suppression of abberant connections during the life-course^53^. Previous research suggests that salience network is one of the latest developing functional networks^54^. Since it is associated with higher-order executive control and decision-making functions, this discovery is supported by the evidence from behavioural studies, which found that higher-order cognitive functions require prolonged development over the life span^55,56^.

Interestingly, several recent studies have proposed that a person's socioeconomic status may influence the differences in their brain's intrinsic neural activity throughout their lifetime^57^.

Gao et al. have found that proxies of socioeconomic status, including higher mother´s education or family income, are associated with better network maturation in the sensorimotor and default mode network, represented in higher matching score with adult forms, higher within-network functional connectivity, or lower outside-network functional connectivity^54^. This study further suggests that children in an enriched environment, largely dictated by socioeconomic status, may acquire better sensory perception/motor coordination (associated with the sensorimotor cortex) and quicker construction of self- consciousness (associated with the default mode network)^54^. Another study has found that higher socioeconomic status was linked to stronger network maturation, particularly pronounced for regions in the limbic, somatomotor, and ventral attention system^57^. Barch et al. found that the amygdala and hippocampus are less functionally connected at rest to a set of cortical regions including the right superior frontal cortex, for children with lower socioeconomic status^58^. Although one may suggest that association between socioeconomic status and network segregation could be caused by genetic differences rather than environmental factors, a recent twin study of adolescents found that the heritability estimate is low and hence, that this relationship might be especially sensitive to environmental influences^59^.

Our results support and supplement these findings to the functional connectivity of salience network. Even though the aforementioned studies imply that other networks may show a similar relationship to parental education and cognitive ability, our study suggests that these associations are specific to salience network. Specifically, we performed additional sensitivity analyses, investigating whether the functional connectivity of the default mode, sensorimotor, visual, dorsal attention, frontoparietal, language or cerebellar network may underlie the association of mother´s education with performance IQ at age 28/29. After correction for multiple testing, we did not find that functional connectivity of any of these networks is associated with both mother´s education and performance IQ at age 28/29 (data not presented in tables). However, it cannot be excluded that these results are falsely negative due to a low sample size. Therefore, future research on larger samples should explore how our findings can be extrapolated to the interaction of several functional networks.

Although laterality of rostral PFC has not been previously discussed to a great extend, Volle et al. have found that lesions in the right rostral PFC were specifically associated with a deficit in time-based prospective memory so time estimation ability was significantly impared in these patients^60^. Patients with left rostral PFC, when engaged with a working memory task, did not make more errors than control subjects, but had the tendency to perseverate with the previous answers^61^. As prospective memory is an integral part of cognitive ability, our work is in accord with Volle et al.^60^, suggesting that changes in functional connectivity of the right rostral PFC are associated with differences in cognitive ability.

This study has several limitations, particularly considering the sample for the analyses in young adulthood. The number of participants is low; therefore, we may have a low power to detect associations. Furthermore, there is selection bias as the participants that remained in the study and became part of the MRI sub-study are better educated themselves and have parents with higher education. However, this sample is unique as it comes from the Czech Republic, a country situated in Central and Eastern Europe, which is underrepresented in research. To the best of our knowledge, our work is the first one to indicate that connections between the rostral PFC and regions responsible for visual data processing in occipital cortex are involved in multisensory data integration and optimisation for a more efficient state, consequently affecting cognitive performance.

## Methods

### Participants

We studied participants of the Czech arm of the European Longitudinal Study of Pregnancy and Childhood (ELSPAC), which has been previously described in detail^62^. Briefly, ELSPAC-CZ is a prenatal cohort whose members were born between 1991 and 1992; their mothers were enrolled between the ultrasound examination at the 20th week of pregnancy and the birth of the child. The parents were asked to fill in several sets of questionnaires regarding themselves and their household, including their education and other markers of socioeconomic circumstances. At age 8, a subset of the cohort participated in a psychological sub-study, during which their cognitive ability was assessed (see details elsewhere^63^). Furthermore, at age 23/24, another sub-study assessed the function and structure of the brain with MRI, as described (see for example^64,65^). Finally, the participants with the neuroimaging data were followed-up to the age of 28/29, when their cognitive ability was assessed. All participants provided a written informed consent and ethical approval was obtained from the ELSPAC Ethics Committee. All methods were performed in accordance with relevant guidelines and regulations and with the Declaration of Helsinki.

### Parental education

Data on mother´s and father´s education is derived from questionnaires administered to the parents at the time of enrollment into the study*.* It was coded in 8 different categories: primary, vocational without high school graduation, vocational with high school graduation, specialized high school with graduation, general high school with graduation, post-high school graduation study, university education and postgraduate education. In the present study, higher value indicates lower education. While the questionnaires completed by the mother included both questions about her own as well as the father´s education, the questionnaires filled in by the father concerned only his own. In case of missing data in the father´s questionnaire, we used the father´s education reported by the mother.

### Cognitive ability

At the age of 8 years, cognitive ability was assessed with the Czech version of the Wechsler Intelligence Scale for Children^66^, third edition. It generates scores on performance IQ (subtests picture completion, picture arrangement, block design, object assembly, coding, mazes, and symbol search), verbal IQ (subtests information, similarities, arithmetic, vocabulary, comprehension, and digit span) and full-scale IQ. At the age of 28/29 years, cognitive ability was assessed using seven—subtest short form^67^ of the Wechsler Adult Intelligence Scale, fourth edition. It allowed generation of performance IQ (subtests picture completion, digit-symbol coding, and matrix reasoning), verbal IQ (subtests information, arithmetic, similarity and digit span) and full-scale IQ.

### Covariates

We considered two measures of socioeconomic circumstances during the first three years of one´s life that could act as confounding factors in the association of parental education with cognitive ability: father´s occupation and household socioeconomic situation. Data on them were collected with questionnaires administered to the parents at 4 different time points: upon enrollment to the study, at 6 months, at 18 months and at 3 years of the child´s life. For items, on which data was available at different time points during the three years, we calculated the arithmetic average from them to decrease measurement error. We re-coded each indicator of early-life socioeconomic circumstances, so that higher values indicate more adverse conditions.

Father´s occupation was coded in the International standard Classification of Occupations (ISCO) 1988 categories. Using the previously suggested algorithm^68^, we recoded the ISCO 88 categories into 10 classes according to the Erikson, Goldthorpe and Portocareros scheme^69^. Household socioeoconomic situation was derived from five variables (household income**,** deprivation**,** basic utilities, household items and crowding ratio) that were first changed to z-scores and then averaged. Household income is the sum of netto incomes of both parents, side incomes as well as social benefits. Deprivation is assessment made by the mother about how difficult it is to secure the family with food, clothes, heating, rent/other fees, and things necessary for the child. Basic utilities are the sum of nine utilities that the participants have at home for their own use: kitchen, flush toilet, running hot water, bathtub, shower, garden/yard, balcony/terrace/loggia, phone, and car. Household items is the sum of owning these ten items: fridge, washing machine, mangle, dishwasher, freezer, microwave, vacuum cleaner, remoska/grill/frying pot, sewing machine and kitchen robot. Crowding ratio was calculated by dividing the number of household members by the number of rooms in the household.

### Functional connectivity of the salience network

At age 23/24, participants underwent MRI of the brain using 3 T Siemens Prisma MRI scanner, which included a structural MRI T1-weighted sequence (voxel size 1 × 1 × 1 mm, 240 slices per slab, TR 2300 ms, TE 2.34 ms, TI 900 ms, and flip angle 8 degrees) and a 7-min closed-eyes resting state functional MRI (fMRI) (voxel size 3 × 3 × 3 mm, TR 2080 ms, TE 30 ms, flip angles 90 degrees, 39 slices, matrix 64 × 64, 200 measurements). Functional connectivity analysis was performed using CONN Functional Connectivity Toolbox and its default pre-processing pipeline^70^. First, functional images were realigned, un-warped, and slice-timing corrected (interleaved bottom-up). Next, the images were co-registered with structural data and spatially normalized to the Montreal Neurological Institute (MNI) space.

Several steps have been undertaken to ensure that the functional connectivity is not impacted by head motion. Potential outlier scans due to subjects´ head movement were assessed using the ARtifact Detection Tools (ART)—based scrubbing. The threshold for potential identification of outliers was set at the 95th percentile in normative samples, a conservative setting for ART. The first-level covariates were realignment, quality assurance time series, the framewise displacement and scrubbing. Finally, the images were smoothed using a Gaussian kernel of 8 mm full width at half maximum and de-noised. Anatomical CompCor (see details^71^) was implemented, extracting a representative noise signal from white matter regions and cerebrospinal fluid, effectively removing outlier scans, using linear regression. Distribution of connectivity values and blood oxygenation level dependent (BOLD) time series were visually checked after de-noising, all participants passed the requirements. The data were band-pass filtered to 0.008 Hz–0.09 Hz.

Seed-to-voxel analysis assessed functional connectivity between the seeds of interest and the rest of the brain, the seeds having been selected based on Harvard–Oxford Structures Atlas. The seeds of interest were seven nodes of the salience network: anterior cingulate cortex (MNI 0; 22; 35); left anterior insula (− 44; 13; 1); right anterior insula (47; 14; 0); left rostral PFC (− 32; 45; 27); right rostral PFC (32; 46; 27); left supramarginal gyrus (− 60; − 39; 31) and right supramarginal gyrus (62; − 35; 32). Pearson’s correlation coefficients were calculated between the seed time course and the time course of all other voxels in the brain. Seed-to-voxel results are reported when significant at a voxel-wise threshold of level of *p* < 0.001 uncorrected and a cluster-level threshold of *p* < 0.05 corrected for FDR. The correlation coefficients were converted to normally distributed scores using Fisher’s transformation.

### Statistical analysis

**Figure 2:**
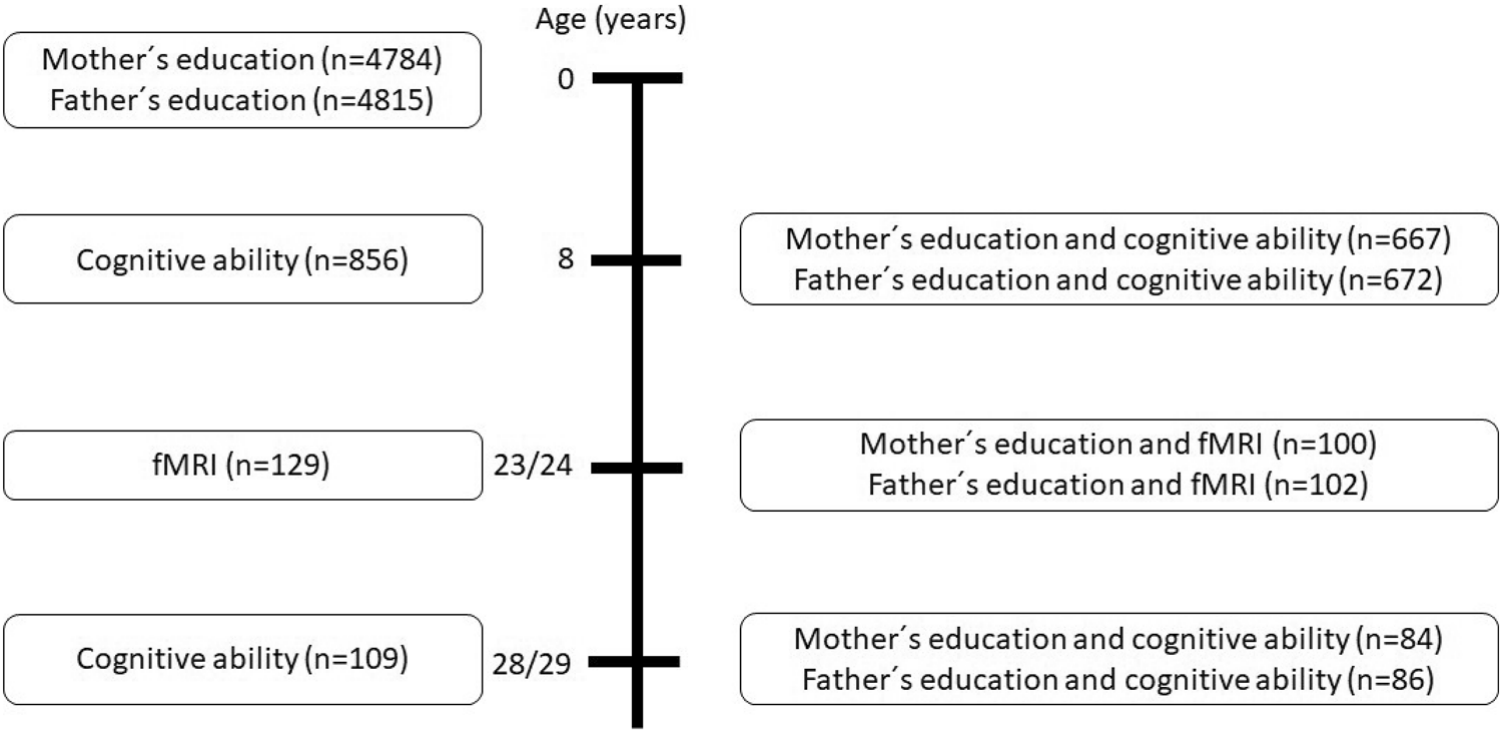
Number of participants in analytical samples.

Derivation of the analytical samples for each analysis is shown on Fig. 2. At age 8, data on cognitive ability and education of parents was available for approximately 670 individuals (667 for mother´s education and 672 for father´s education). At age 23/24, approximately 100 people had data on fMRI and education of parents (100 for mother´s education and 102 for father´s education). At age 28/29, information on cognitive ability and education of parents was available for 84 individuals in case of mother´s education and 86 individuals in case of father´s education. Data is presented as mean ± standard deviation, median and interquartile range or frequency (n; %), where appropriate. First, we used Spearman´s nonparametric correlations to assess relationships between parental education (separately for mother´s education and father´s education) and cognitive ability at age 8 (separately for verbal IQ, performance IQ, full-scale IQ). Second, we applied linear regression to estimate B with 95% CI for the association of parental education (mother´s and father´s education entered simultaneously into the model as independent variables) with cognitive ability at age 8 (dependent variable; separate models for verbal IQ, performance IQ, full-scale IQ), adjusting for sex, father´s occupation and household socioeconomic situation. Third, we repeated the two previous steps for the outcome cognitive ability at age 28/29 (separately for verbal IQ, performance IQ, full-scale IQ). The fit of the models was assessed for homoscedasticity and normality of the error distribution.

Fourth, we investigated whether functional connectivity of the salience network may be a biomarker of the association of parental education with cognitive ability. As neuroimaging was not performed in childhood, before cognitive ability was measured at age 8, but only in young adulthood (at age 23/24), we only studied the association of functional connectivity of the salience network with cognitive ability at age 28/29. Specifically, we transferred the coefficients for clusters of voxels showing a significant correlation with parental education from CONN into SPSS and assessed their relationship with cognitive ability at age 28/29, using Spearman´s nonparametric correlations. We corrected the p value for the number of tested clusters, using Benjamini–Hochberg method. We assessed collinearity with variance inflation factor (VIF), which reports how much the variance of the estimated coefficients increases is due to collinear independent variables. Specifically, VIF reports how much of a regressor´s variability is explained by the rest of the regressors in the model due to correlation among those regressors^72^. Suggestions for a cutoff point, above which VIF would be too high, are 5 or 10^72^. As VIF was lower than 3 in all models, we kept all covariates in the final models. We also tested, whether sex is an effect modifier, by including an interaction between sex and parental education. Likelihood ratio test assessed the interaction effect.

## Data Availability

Access to the data is provided free of charge to researchers upon reasonable request. More information can be found on this website: elspac.cz. The study protocol and syntax of the statistical analysis will be shared upon request from the corresponding author of this study.
